# Visual Space Constructed by Saccade Motor Maps

**DOI:** 10.3389/fnhum.2016.00225

**Published:** 2016-05-18

**Authors:** Eckart Zimmermann, Markus Lappe

**Affiliations:** ^1^Cognitive Neuroscience (INM3), Institute of Neuroscience and Medicine, Research Centre JülichJülich, Germany; ^2^Institute for Psychology, University of MünsterMünster, Germany

**Keywords:** saccades, saccade adaptation, visual space, mislocalization, efference copy

## Abstract

How visual space is represented in the brain is an open question in neuroscience. Embodiment theories propose that spatial perception is structured by neural motor maps. Especially, maps which code the targets for saccadic eye movements contain a precise representation of external space. In this review article, we examine how modifications in saccade maps are accompanied by changes in visual space perception. Saccade adaptation, a method which systematically modifies saccade amplitudes, alters the localization of visual objects in space. We illustrate how information about saccade amplitudes is transferred from the cerebellum (CB) to the frontal eye field (FEF). We argue that changes in visual localization after adaptation of saccade maps provide evidence for a shared representation of visual and motor space.

## Introduction

The idea that action and perception are interdependent, or that the former shapes the latter, has a long tradition in neuroscience. It dates back to the ideomotor theories of Lotze (1852) and James (1890), which posited that cognitive metrics (e.g., visual space), are represented relative to intentions to move. Later prominent examples include Gibson’s (1979) ecological approach to perception, in which the basic unit of perception are affordances or possibilities for action, Prinz’s (1984) common coding theory, which claims a shared representation for perception and action, and the sensorimotor contingencies account, which states that our perceptual experience of the world is composed of the sensorimotor transformation laws that govern how we interact with the world (O’Regan and Noë, 2001). However, the question of how space is represented in the brain is still left unanswered.

In principle, early visual areas with their retinotopic organization might seem well suited to map external space isomorphically. However, several factors discredit the supposed retinotopic topography as an accurate mirroring of external space. First, the distortion and blur of the retinal image by spherical and chromatic aberration of the crystalline lens and second, the magnification of the foveal area in cortex lead to a rather heterogenous cortical representation of space (Wolff, 2004). Other distortions occur through neural adaptation processes constantly taking place in early visual areas (Clifford et al., 2000). A map which is so malleable to several kinds of distortions is therefore an unlikely candidate to deliver a consistent representation of visual space. Consistency, however, is required to produce the precision of oculomotor behavior. Instead of reading out spatial information from visual maps directly, another possibility is to use a code of visual position from motor maps In fact, the most precise and consistent information on the position of a visual object is needed only when one wants to act upon that object, for example to grasp it or to look at it. Saccade motor maps necessarily contain an accurate representation of saccade target locations, given the precision of saccade landing positions (Kowler, 2011). A shared position code for perception and action would save computational resources since only one rather than two separate maps would be needed. This also avoids the problem of aligning the maps for visual and motor space.

In this review, we illustrate an approach to test the hypothesis of a shared map for motor and visual space. This approach involves the experimental induction of short-term modifications in the metrics of saccade motor maps in order to observe whether these changes are followed by distortions in visual space. Saccade adaptation is a method which modifies the amplitude of saccade eye movements (for reviews see Hopp and Fuchs, 2004; Pèlisson et al., 2010; Herman et al., 2013). This kind of oculomotor plasticity can involve multiple areas in the brain. We will first discuss studies which distinguish the different contributions of subcortical and cortical regions to saccade adaptation and then provide evidence demonstrating that changes in the oculomotor maps are accompanied by changes in the visual localization of objects in space.

## Visual Effects of Saccade Adaptation

**Figure 1:**
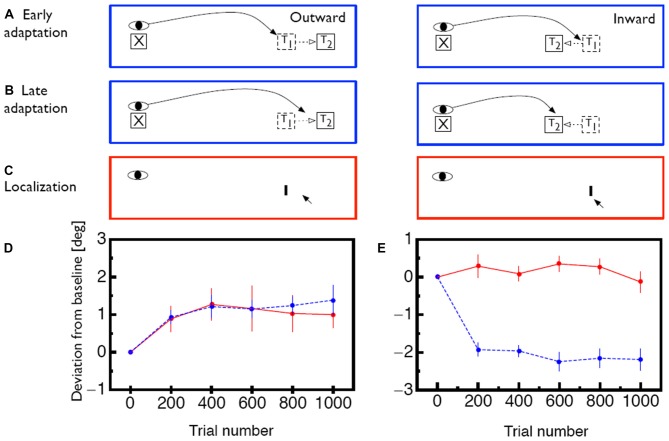
**(A)** Illustration of the saccade adaptation paradigm. At the beginning of a trial the eye is directed to a fixation point (X). Then, a saccade target (T_1_) appears and the saccade is initiated. While the saccade is in flight, the target is displaced to a new position (T_2_) in either outward or inward direction.** (B)** After several adaptation trials, the oculomotor system adapts to the intrasaccadic target displacement. The saccade now lands closer to the displaced position T_2_ even though the saccade target is initially shown at position T_1_. **(C)** lllustration of how visual mislocalization was measured. Subjects were asked to keep gaze fixated during the whole trial. Localization was measured in complete darkness. A probe stimulus (I) was briefly flashed and subjects indicated its apparent position with a mouse pointer. **(D)** Time course of saccade amplitude change (blue) and apparent probe location change (red) for outward adaptation. Error bars represent SEM. **(E)** Same for inward adaption. Error bars represent SEM. Data shown in **(D,E)** is replotted from Zimmermann and Lappe (2010).

The oculomotor system constantly monitors the accuracy of executed saccades and compensates systematic errors between intended and actual saccade landing positions (Robinson, 1975). With the experimental paradigm called saccade adaptation, this compensation mechanism can be triggered artificially in the laboratory (Figure 1A): subjects are asked to perform a saccade to a target. While the saccade is in flight the target is displaced by a specific amount and in a specific direction (McLaughlin, 1967). Subjects mostly remain unaware of the displacement since visual sensitivity is drastically reduced during saccade execution (Bridgeman et al., 1975). After saccade landing the oculomotor system detects the mismatch between the planned landing position and the physical location of the target. As a consequence, the saccades of the subsequent trials are modified in amplitude to reach the displaced target position more accurately (Figure 1B). Saccade adaptation develops gradually across trials, usually following an exponential learning curve (Figures 1D,E, blue lines). In humans it reaches an asymptotic level within 30–60 trials (Deubel et al., 1986; Deubel, 1987; Frens and van Opstal, 1994; Albano, 1996; Watanabe et al., 2003). These changes are long-lasting and can be measured even several days after induction (Alahyane and Pélisson, 2005; Wang et al., 2012). Saccade adaptation is selective for the direction and amplitude of the adapted saccades and transfers only to saccades with sufficiently similar amplitudes (Frens and van Opstal, 1994; Collins et al., 2007). This limited range of transfer has been termed the adaptation field. Additionally, saccade adaptation is specific to the orbital position of the eyes during the induction phase. Adaptation magnitude decreases if the eye position is changed between induction and test period (Shelhamer and Clendaniel, 2002a,b; Alahyane and Pélisson, 2004; Zimmermann and Lappe, 2011). Saccadic adaptation is not specific for color and shape of the saccade target (Deubel, 1995b). The temporal frequency of flickering targets, however, has been reported to act as a contextual cue (Herman et al., 2009). Specifics of the time course of adaptation (Ethier et al., 2008; Xu-Wilson et al., 2009) and of the dynamics of adapted saccades (Chen-Harris et al., 2008; Ethier et al., 2008) have been interpreted to distinguish two types of adaptation.The first adapts motor performance via changes to internal monitoring in a forward model of the eye movement. The second changes the motor command, i.e., the target representation.

To test whether saccade adaptation changes spatial perception, several studies asked subjects to localize probe objects which were presented briefly before the initiation of an adapted saccade. Subjects had to report the location of the probe after they had performed the adapted saccade (Awater et al., 2005; Bruno and Morrone, 2007; Collins et al., 2007; Georg and Lappe, 2009; Zimmermann and Lappe, 2009). In these studies objects were mislocalized in the direction of adaptation. In order to check the role of visual references for localization, the saccade target was switched off during saccade execution on a portion of trials. Significant mislocalization occurred both when the saccade target remained visible as well as when it was switched off. Thus, the mislocalization was not due to the intrasaccadic step which might have acted as a visual landmark. However, since in these experiments an adapted saccade was performed between presentation and localization of the probe stimulus, the question remained whether saccade adaptation distorts the representation of visual space or whether the mismatch between expected and actual landing position produced the mislocalization. To answer that question localization needs to be tested when the eye is fixating.

The first study which tested changes to spatial perception following saccade adaptation during ocular fixation was reported by Moidell and Bedell (1988). After rightward saccade adaptation they asked subjects to judge the distance of a stimulus shown at the adapted location while keeping gaze directed at the fixation point. Subjects had to estimate the distance between the stimulus and the fixation point relative to the distance between fixation point and a reference stimulus shown in the unadapted opposite hemifield. With this task only small shifts in visual perception were found (around 0.5°), which were significant only when inward and outward adaptation were contrasted. However, localization of visual stimuli can principally be performed in two ways: either allocentrically, where the distance of the probe stimulus to a reference object is used or egocentrically, where the absolute spatial position is used (Müsseler and van der Heijden, 2004). The task used by Moidell and Bedell (1988) inherently required subjects to localize the probe allocentrically, relative to the fixation point.

We assumed that saccade adaptation might have changed coordinates in egocentric localization. To test effects of saccade adaptation on egocentric localization we implemented a localization task which disabled any possibility for allocentric localization (Zimmermann and Lappe, 2010; Figure 1C). Reference objects were removed by conducting the experiment in a completely dark room. Subjects were adapted in either inward or outward direction. Interspersed in the adaptation trials were blocks of localization trials. In these trials the fixation point was switched off and subjects had to keep gaze at its remembered position. A probe stimulus was presented for 20 ms at the adapted location. Briefly afterwards a mouse cursor appeared which subjects had to use to indicate the perceived probe position. We found shifts in the perception of visual space which were as large as saccade adaptation magnitude when subjects were adapted in outward adaptation (Figure 1D). When subjects were adapted in inward direction however, no mislocalization of the probe stimulus occurred (Figure 1E). Similarly, a study by Hernandez et al. (2008) found transfer of saccade adaptation to hand pointing movements and in agreement with our argumentation these authors found shifts in hand pointing only after saccade adaptation in outward but not in inward direction.

Outward adaptation takes more time to develop and is less complete than inward adaptation (Miller et al., 1981; Semmlow et al., 1989; Straube and Deubel, 1995; Straube et al., 1997; Ethier et al., 2008; Hernandez et al., 2008; Cecala and Freedman, 2008; Panouillères et al., 2009; Zimmermann and Lappe, 2010; Schnier and Lappe, 2011, 2012; Mueller et al., 2012). This implies that over the course of trials the visual error between saccade landing and post-saccadic target position is larger in outward than in inward adaptation. We assumed that the cumulative amount of visual error might induce shifts in the space map. Earlier studies had already suggested that the visual error is an important factor in driving saccade adaptation (Wallman and Fuchs, 1998).

**Figure 2:**
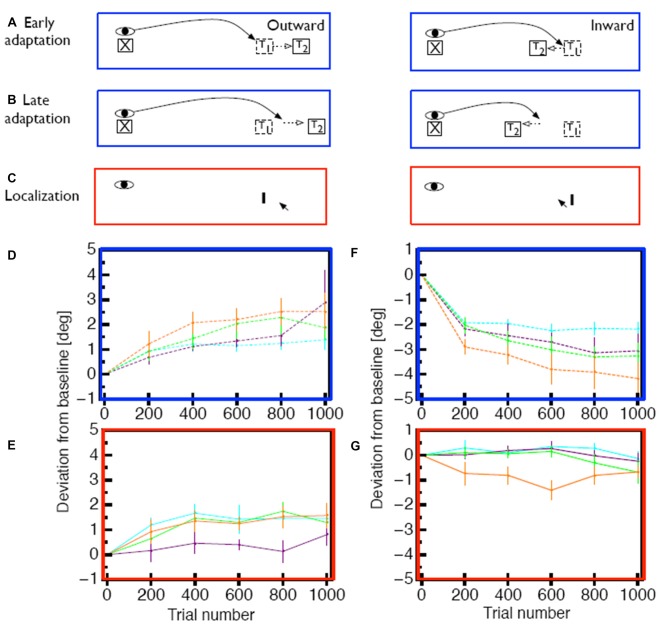
**(A)** Illustration of the constant error paradigm. In this paradigm the target was shifted such that the visual error between landing position and target was constant across trials. **(B)** After several adaptation trials saccades adapt in response to the post-saccadic visual error, but the visual error remains the same. **(C)** Measurement of apparent probe position was the same as in Figure 1. **(D)** Time course of saccade amplitude change in the constant error paradigm for 1° (purple), 2° (green)and 3° (orange) outward visual error. As a comparison, saccade amplitude changes form the constant target shift (3°) paradigm are shown in cyan. Error bars represent SEM. **(E)** Change in apparent probe location after outward adaptation. **(F)** Time course of saccade amplitude change in the constant error paradigm for 1° (purple), 2° (green)and 3° (orange) inward visual error.** (G)** Change in apparent probe location after inward adaptation. Data shown in **(D–G)** is replotted from Zimmermann and Lappe (2010).

To test the hypothesis that the size and persistence of the visual error is responsible for the mislocalization magnitude, we used a saccade adaptation variant (Robinson et al., 2003) in which the saccade landing position is predicted from online eye position data and the target is stepped to a location that is a constant, pre-determined distance from the landing position of the saccade (Figures 2A–C). With this method it is thus possible to apply a constant visual error for either inward or outward adaptation in each trial (Figures 2D,F). For outward adaptation mislocalization was observed after adaptation to comparably big visual errors of 2° and 3° but not after adaptation of to small visual errors of 1° (Figure 2E). Moreover, in this paradigm we also found clear mislocalization effects for inward adaptation (Figure 2G) but also only for the largest visual error tested (3°). This experiment hence showed that saccade adaptation modifies space perception but that the amount of modification of space perception depended on the size of the visual error, as well as its persistence.

Further evidence for a modification of space perception during fixation was provided by Garaas and Pomplun (2011). They adapted selectively either the horizontal or the vertical component of many different saccades. Before and after adaptation observers had to compare the lengths of the vertical and the horizontal line of a cross. Adaptation of the vertical component of saccades induced misjudgments of the vertical line length. After outward adaptation vertical lines were perceived as longer and after inward adaptation as shorter. Similarly, after horizontal inward adaptation horizontal lines appeared shorter. These distortions occurred even for objects that were continuously presented during fixation. Khan et al. (2010) showed that after saccade adaptation the facilitating effect of attentional cuing is strongest at the adapted not the physical saccade target location. This finding suggests that attention is informed about adaptation or even rely on a shared representation between sensory and motor space and attention.

## Forward Model in Cerebellum and Target Representation in FEF

To understand how saccade adaptation modifies space perception we need to ask how and where in the brain the common metric for saccades and spatial perception may reside. For this question, the different ways in which saccade adaptation can occur are important.

Because of the high velocity of a saccade, control of its trajectory cannot rely on ongoing visual feedback but must use feedforward signals generated by a forward model of saccade kinematics. Forward models, in general, compute predictions of the outcome of an action based on an action command. The forward model of saccade kinematics is hypothesized to monitor eye trajectory during each ongoing saccade and correct amplitude by slowing the eye if it is moving too fast or speeding the eye up if it is moving too slow, based on a signal of the intended amplitude. An optimal control model of saccades demonstrated that the time course of adaptation (Ethier et al., 2008; Xu-Wilson et al., 2009) and of the dynamics of adapted saccades (Chen-Harris et al., 2008; Ethier et al., 2008) distinguished between changes in this forward model of saccade trajectory and changes in the motor command, i.e., the target representation. The probability to assign errors to the target representation increases with increasing post-saccadic error. Ethier et al. (2008) proposed the Cerebellum (CB) to contain the forward model and suggested that the changes in the motor command occur in the superior colliculus (SC). The view that the CB contains forward models for motor learning is well-established (for a review see Ito, 2013). In humans it has been found that an intact CB is necessary for adaptation (Straube et al., 2001; Alahyane et al., 2008; Choi et al., 2008; Golla et al., 2008; Panouillères et al., 2013). Electrophysiological and lesion studies in nonhuman primates have shown the involvement of the oculomotor vermis of the CB (Takagi et al., 1998; Barash et al., 1999; Robinson et al., 2002; Catz et al., 2005, 2008). Neuroimaging studies (Desmurget et al., 1998; Blurton et al., 2012) and studies using repetitive transcranial magnetic stimulation (Jenkinson and Miall, 2010) or transcranial direct current stimulation (Panouillères et al., 2015) over the posterior vermis confirmed the involvement of the vermis in saccade adaptation. Hence, the adaptation of saccade trajectory via a change in the forward model of saccade trajectory is likely a cerebellar function.

The mechanism and the neural substrate of the second type of adaptation, that of the target representation, is less clear, but it might also involve a prediction of saccade outcome based on the saccade motor command by a forward model of the visual consequences of the saccade. In this view, a prediction about the post-saccadic visual error would be generated before saccade initiation and compared to the actual image obtained after landing. A mismatch in this comparison would induce adaptation to minimize the difference. Indeed, studies have reported evidence that saccade adaptation relies on a comparison between the predicted and the actual post-saccadic retinal error (Bahcall and Kowler, 1999; Chen-Harris et al., 2008; Collins and Wallman, 2012; Wong and Shelhamer, 2012; Herman et al., 2013). The question, then, is in which neural structures the prediction is converted into a change of the target representation.

Most research on this question concentrated on the SC, but its involvement in saccade adaptation is still debated. Movement fields of neurons in the SC show no changes during adaptation of reactive saccades (Frens and Van Opstal, 1997; Quessy et al., 2010), although changes in firing rates have been observed (Takeichi et al., 2007). Two studies (Kaku et al., 2009; Soetedjo et al., 2009) have delivered subthreshold electrical stimulation to the SC which signaled an apparent error in saccade landing. After several trials, saccades adapted to reduce the apparent error, suggesting that activity in the SC may provide the error signal that drives adaptation. It is important to note, however, that with present recording techniques, it is difficult to answer definitively whether coding in SC changes during adaptation. Because the SC relies on a population code with strong local inhibition, it is possible that subtle changes in firing rate as observed by Takeichi et al. (2007) might produce adapted saccades without changing the overall structure of individual movement fields. To definitively answer this question, one would need to simultaneously record from neurons with receptive and motor field centers spread over a large area, which is at present possible only in cerebral cortex and impossible in a deep structure such as the SC.

A participation of the parietal cortex in saccade adaptation has been reported by a recent fMRI study (Gerardin et al., 2012). This study showed that scanning saccade adaptation involves dorsal areas of the frontal and parietal cortex whereas reactive saccade adaptation involves more ventral parts of the frontal and parietal cortex. Causal evidence for a role of the parietal cortex in saccade adaptation has been provided by Panouillères et al. (2012). They adapted reactive and voluntary saccades while single-pulse transcranial magnetic stimulation (spTMS) was applied over the posterior intraparietal sulcus. The stimulation impaired voluntary saccade adaptation when spTMS was applied 60 ms after saccade initiation. Reactive saccade adaptation was impaired when spTMS was applied 90 ms after saccade onset. However, it is not clear whether the parietal cortex contribution to adaptation involves a change in saccade targeting or, instead, the processing of the error signal. Steenrod et al. (2013) recorded activity from single neurons in the lateral intraparietal area in the monkey and found that movement fields were unchanged after inward saccade adaptation. These results suggest that the parietal cortex is uninformed about saccade inward adaptation. Since, however, changes in space perception in humans (Zimmermann and Lappe, 2010) and monkeys (Gremmler et al., 2014) are seen predominantly during outward adaptation the possibility that the parietal cortex contributes to these changes is still open.

**Figure 3:**
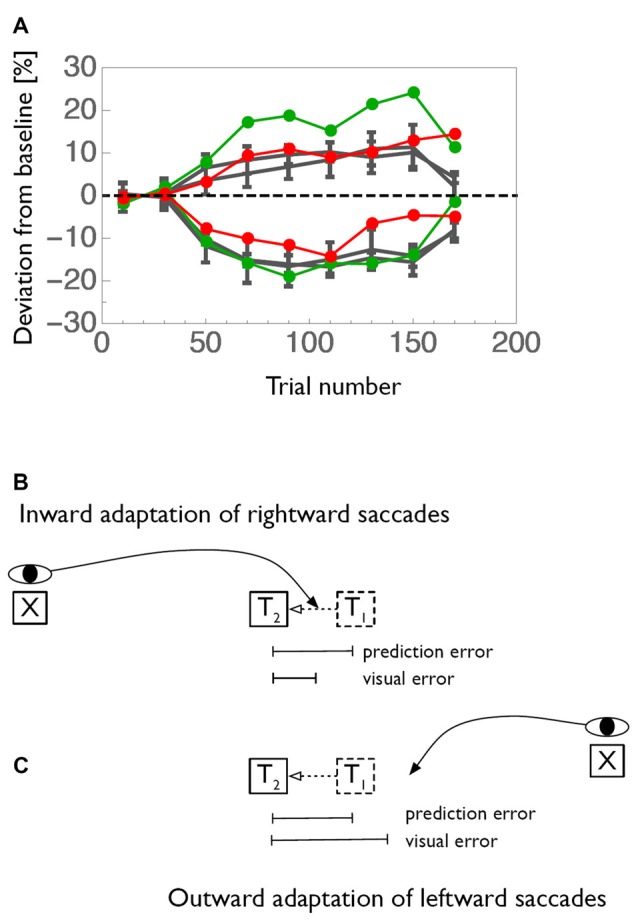
**(A)** Adaptation curves for inward (negative values) and outward (positive values) adaptation of a patient with a lesion in the right ventrolateral nucleus (VL) of the thalamus. Rightward saccades of the patient are shown in red and leftward saccades in green. Average adaptation curves of an age-matched control-group is shown in gray. **(B)** Graphical illustration of how prediction error and visual error contribute to saccade adaptation. Usually, saccades undershoot their target. Thus, when the target is shifted in inward direction, the saccade will land between the initial and the shifted position of the target. The visual error between landing position and shifted target location is therefore smaller than the error between predicted and shifted target position. **(C)** Conversely, when the target is displaced in outward direction the visual error becomes larges than the prediction error. Data shown in **(A)** is replotted from Zimmermann et al. (2015).

Further candidate for modification of the saccade target command are the eye fields in the frontal cortex, i.e., the frontal (FEF) and supplementary (SEF) eye fields Changes in functional MRI (fMRI) BOLD activity during saccade adaptation have been reported in these areas (Blurton et al., 2012; Gerardin et al., 2012). Single unit electrophysiology data is lacking. The frontal cortex is the recipient of a feedback pathway from the CB through the ventrolateral nucleus (VL) of the thalamus. Two studies (Gaymard et al., 2001; Zimmermann et al., 2015) tested patients with lesions in the VL. These patients exhibited a strong impairment of inward saccade adaptation contraversive to the lesioned side and, surprisingly, a larger-than-normal outward adaptation of saccades towards the ipsilesional side (Figure 3A). These results demonstrate the involvement of cortical areas in adaptation and are consistent with the idea that saccade adaptation relies on a a comparison between the predicted and the actual post-saccadic retinal error (Bahcall and Kowler, 2000; Chen-Harris et al., 2008; Collins and Wallman, 2012; Wong and Shelhamer, 2012; Herman et al., 2013). In this view, the pathway from the CB through the thalamus would carry the predicted retinal error to make it available in cortical areas for a comparison to the actual error.

**Figure 4:**
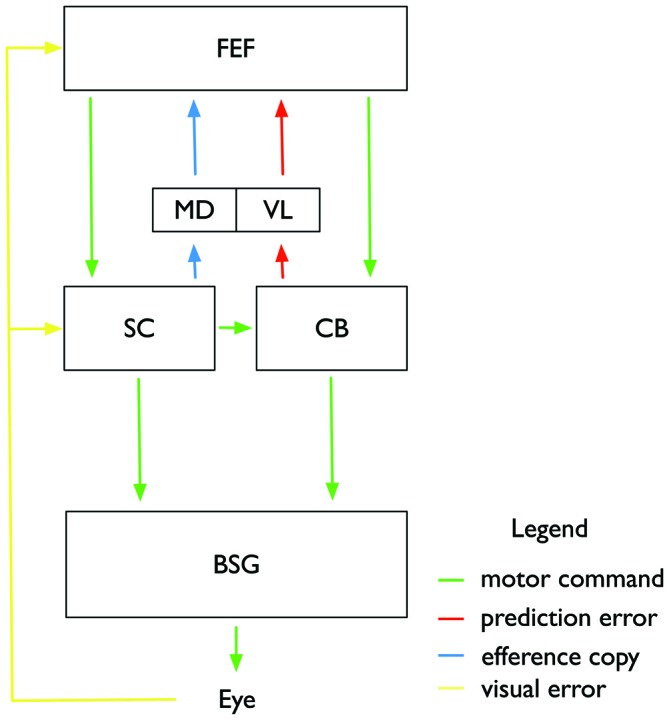
**Illustration of the proposed subcortical and cortical circuitry involved in controlling saccade adaptation and associated changes in visual space perception.** Motor commands (green color) are sent from the frontal eye fields (FEF) to the superior colliculus (SC) and the Cerebellum (CB), and from there to the brainstem saccade generator (BSG) which controls the eye muscles. Adaptation of the feedforward pathway takes place in the CB. A feedback pathway from the CB to the FEF via the VL of the thalamus carries a prediction signal about the current adaptation state in the CB and the expected post-saccadic target location. An efferency copy of the intended saccade is sent from the SC through the mediodorsal nucleus (MD) to the FEF. FEF and SC also receive post-saccadic visual information about the visual error between actual saccade landing and shifted target position. Adaptation of the motor command in FEF affects both saccade amplitude and perceptual space.

Figure 3B illustrates how both the comparatively small inward adaptation for contraversive (leftward) saccades and the comparatively large outward adaptation for ipsiversive (rightward) saccades can be explained by a deficient prediction of saccade endpoints. The first important point is that saccades of the size tested in this experiment are typically hypometric, i.e., they fall short of the target. This was the case also for the patient. Normally, when the prediction of the saccade is available, the saccade undershoot is anticipated and the target is expected to lie somewhat away from the fovea after saccade landing. The mismatch between the predicted target position and the actual target position in the adaptation paradigm, i.e., the prediction error, is then equal to the target shift. This is true both for inward and for outward adaptation. In contrast, when the prediction signal is unavailable, as we propose for the patient, then the error signal driving adaptation cannot take the predicted target location into account and instead has to rely on the post-saccadic distance of the target from the fovea, i.e., the visual error. Because of the typical hypometria, for inward adaptation of leftward saccades the visual error is actually smaller than the prediction error. Since adaptation is influenced by the size of the error signal, this condition results in a smaller-than-normal adaptation. In contrast, for outward adaptation of rightward saccades, the hypometria produces a visual error signal that is larger than the prediction error. Hence, if the adaptation process has no access to the prediction signal the adaptation will be stronger than normal. Both effects are seen in the patient. However, inward adaptation of rightward saccades and outward adaptation of leftward saccades appear normal in this patient. The common aspect of the two conditions in which differences in adaptation occur is the direction of the target shift (Figure 3C). In both cases, the target shift is to the left, i.e., contralateral to the lesion site. Hence, we propose that the feedback pathway carries a prediction error signal for contralateral errors, consistent with the contralateral representation of saccade targets in the recipient cortical area (red arrows in Figure 4).

We have described above that visual effects following saccade adaptation depend on large and consistent visual errors. Following the predictions from the optimal control model (Chen-Harris et al., 2008; Ethier et al., 2008) we assume that small retinal errors will be corrected by the CB. Retinal errors, however, which deviate too strongly from their prediction will activate adaptive processes in cortical areas (Figure 4). We suggest that adaptive changes of the target representation may occur in frontal cortex. The FEF, for example, contains a map of visual and motor space (Bruce and Goldberg, 1985). Its many reciprocal connections to visual areas enable it to provide visual feature representations with spatial metrics (Huerta et al., 1987; Baizer et al., 1991; Schall et al., 1995; Stanton et al., 1995; Barone et al., 2000). Thus, if part of saccade adaptation occurs in the FEF, this could explain the simultaneous effects on saccade amplitude and on visual localization. The negative findings regarding the involvement of the SC or parietal cortex would be consistent with our view if saccade adaptation is controlled by the route from the FEF to the CB.

## Efference Copy

Reception of subcortical saccade signals in the FEF to compare them to pre-saccadic predictions is discussed as a mechanism to solve the problem of visual stability (Sommer and Wurtz, 2004). Every time we move our eyes, the retinal coordinates shift relative to the coordinates of external space. To ensure that this displacement is not interpreted as a movement in external space, areas representing visual space must be informed about the eye movement. A signal, variously called “efference copy” (von Holst and Mittelstaedt, 1950) or “corollary discharge” (Sperry, 1950) has been postulated to carry eye movement information from motor to visual areas. This signal would encode the size and direction of the upcoming saccade, thus enabling visual areas to predict the retinal displacement. Sommer and Wurtz (2004) identified a pathway from the SC through the thalamus to the FEF (Figure 4) which might transport the suggested efference copy signal. This pathway has recently been linked to visual stability in humans (Ostendorf et al., 2010) and monkeys (Cavanaugh et al., 2016) When the thalamic MD feedback path was inactive (due to a lesion in a human patient and by experimental inactivation in the monkey), displacement discrimination performance became inaccurate. The importance of the efference copy—encoding saccade amplitudes—for visual space becomes relevant also for the interpretation of the visual effects accompanying saccade adaptation. Saccade adaptation shifts actual saccade landing positions relative to the intended landing location. The efference copy can then either be informed about the adaptation or uninformed. The latter would be the case if the neural locus of adaptation is independent of the efference copy pathway. Some researchers have claimed that visual effects observed after saccade adaptation are the result of an efference copy uninformed about adaptation. Bahcall and Kowler (1999) suggested that the mislocalization occurs because the feedback about the executed eye movement, i.e., an efference copy signal, is unaware of adaptation. Visual areas assume that saccade landing was correct and therefore, compensate the retinal displacement with the size of the intended, not the actual saccade, resulting in mislocalization. This explanation however faces two difficulties: first, mislocalization should in this view occur only if an adapted saccade is executed. However, as described above, several studies have now reported adaptation-induced mislocalization during ocular fixation. Second, it would predict a uniform shift of mislocalization over the whole visual field. But this is not the case: Mislocalization is restricted to the spatial adaptation field surrounding the adapted saccade (Collins et al., 2007). Moreover, a recent study has advanced the view that there is an accurate efference copy matching the performed saccade amplitude also for adapted saccades (Collins, 2010).

Another finding stands in contrast to an account of assuming one unitary efference copy signal: adaptation is specific for saccade types: reactive saccades, which are driven by a sudden onset of the saccade target, are independently adaptable from voluntary saccades, in which the saccade targets are presented continuously and saccades are performed by the subject in a self-paced manner (Erkelens and Hulleman, 1993; Deubel, 1995a; Fujita et al., 2002; Hopp and Fuchs, 2004; Collins and Doré-Mazars, 2006; Cotti et al., 2007). If saccades of one type are adaptively changed, the adaptation transfers only partly to the other type, suggesting the involvement of different neural mechanisms (Alahyane et al., 2007, 2008; Cotti et al., 2009; Schnier and Lappe, 2012). Important to the question of the efference copy signal is the observation that mislocalization is selective for the types of saccades adapted and the temporal properties of stimuli that have to be localized (Zimmermann and Lappe, 2009). Adaptation of reactive saccades induced mislocalization of flashed probes and adaptation of scanning saccades induced mislocalization of flashed and stationary probes. It might therefore be too simplistic to speculate about a singular efference copy. Instead, one may assume that several efference copies are generated for each saccade by various structures involved in saccade initiation, and that some of these signals (for example that from SC to FEF) reflect the unadapted saccade whereas others (for example that from the CB to the FEF) provide an accurate estimate of the adapted saccade. We therefore suggest that mislocalization for objects shown before and localized after an adapted saccade is the combination of two effects: The effect of adaptation on visual space and the mismatch between an unadapted efference copy signal and the physical post-saccadic input.

## Integration of Visual Features Across Saccades

Saccadic adaptation, we propose, acts as a way to calibrate visual space perception by observing and correcting mismatches between the peripheral view of a target and the central view of that same target after a saccade towards it. Similar trans-saccadic calibration procedures might exist also for other visual qualities.

Visual perception always appears stable and coherent although the distribution of receptors is heterogeneous within the retina. In the classical “pure vision” account, this would pose the need for compensation mechanisms constantly adjusting spatial relationships across receptor inhomogeneities. In sensorimotor theories, the inhomogeneities become an integral part of space perception. Knowing how the same rectangle looks when seen in the fovea compared to when seen in the periphery means also knowing where in space the rectangle is located. When a target is initially seen in the periphery, it activates a relatively small number of retinal ganglion cells whereas after an eye movement that brings the target to the fovea, it activates a far larger number of retinal ganglion cells. Due to the different distribution of receptors in periphery and fovea one might think that the object’s perceived size should vary across saccades. However, such trans-saccadic changes in size are never observed (visual constancy). Learning of sensorimotor contingencies is likely responsible for associating how the same object looks in the fovea and in the periphery. If this is the case, it should be possible to establish new associations by inducing trans-saccadic feature changes. Thus, similar to changing position in the saccadic adaptation paradigm, other features as spatial frequency or size could also be manipulated. Indeed, Herwig and Schneider (2014) trained participants with new feature associations by changing the spatial frequency of a grating trans-saccadically. After learning participants performed a visual search task in which behavior was biased toward previously associated presaccadic peripheral input. Bosco et al. (2015) either increased or decreased the size of an object during the execution of a saccade. Not only the saccade amplitude but also the perceptual estimate of object size changed over the course of object changes. Similar results were found by Valsecchi and Gegenfurtner (2016). These studies therefore suggest that saccade contingencies not only interact with our estimation of visual space but more generally with our perception of object features. Adaptations of size and feature perception across saccades are not easily explainable from a simply efference copy mechanism of saccade amplitude. In a broader, view, however, they are compatible with the hypothesis that prediction of trans-saccadic retinal changes are used for calibrating spatial perception. Much as the efference copy is used to predict the location of the target after the saccade it could also be used to generate a prediction of which object will be in foveal view after the saccade. If this prediction includes the features of the object, as seen in peripheral view, and if these features in foveal view do not match the prediction, then a recalibration of the peripheral feature representation may be induced. In neural terms, this might consist of feedback of prediction error for object features or size to areas in the ventral stream of visual cortical processing.

## Summary

We conclude first that saccade adaptation changes the perception of visual space and second that visual space is based on an oculomotor map. We have reviewed findings which report visual mislocalization following saccade adaptation both after an adapted saccade was performed and during ocular fixation. We have argued that adaptation takes place in a neural map which structures visual and motor space with the same coordinates. Shared coding would be advantageous not only from the perspective of computational resources but also for the alignment of visual and motor space. The FEF is a likely candidate for this space map because it contains the topographic architecture necessary for saccade planning and wide connections to visual areas.

## Author Contributions

EZ and ML wrote the manuscript.

## Conflict of Interest Statement

The authors declare that the research was conducted in the absence of any commercial or financial relationships that could be construed as a potential conflict of interest.
